# Rapid assessment of pre-service midwifery education in conflict settings: findings from a cross-sectional study in Nigeria and Somalia

**DOI:** 10.1186/s12960-025-00977-6

**Published:** 2025-02-03

**Authors:** Emilia Iwu, Shatha Elnakib, Hawa Abdullahi, Rejoice Helma Abimiku, Charity Maina, Asia Mohamed, Kazeem Olalekan Ayodeji, George Odonye, Rifkatu Sunday, Maryan Abdulkadir Ahmed, Mohamed Ahmed Omar, Abdirisak A. Dalmar, Emilie Grant, Hannah Tappis

**Affiliations:** 1 Institute of Human Virology, Abuja, Nigeria; 2 Rutgers University School of Nursing, Newark, NJ USA; 3 Johns Hopkins Bloomberg School of Public Health, Johns Hopkins University Center for Humanitarian Health, Baltimore, USA; 4 Somali Research and Development Institute, Mogadishu, Somalia

**Keywords:** Midwifery, Conflict, Somalia, Nigeria, Pre-service education

## Abstract

**Background:**

There is a dearth of evidence on the scale, scope and quality of midwifery education programs in conflict-affected settings. This study sought to assess the extent to which midwifery pre-service education programs meet national and global standards, and to explore how conflict affects pre-service midwifery education in Yobe State Nigeria and the Benadir and Galgaduud regions of Somalia.

**Methods:**

A rapid assessment of midwifery education programs was conducted in the two midwifery education programs in Yobe State, Nigeria and in seven purposively selected programs in Somalia using an adaptation of the Midwifery Education Rapid Assessment Tool. Information was collected through interviews with program leadership, teachers, students, and clinical preceptors during school and clinical practice site visits. Researchers adapted the tool to reflect national and international standards, incorporating supplementary questions to capture considerations specific to conflict-affected contexts. Data were analyzed by program and country using Stata and Excel.

**Results:**

In Nigeria, each school met 17 and 18 standards, respectively, out of 22 across assessment domains (77.3%–81.8%). In contrast, in Somalia, schools met between 6 and 10 standards out of the 18 standards for which data were available (33.3%–55.6%). The biggest gaps in Somalia were in leadership, infrastructure and resources. No schools in either setting had sufficient space or clinical practice sites that met International Confederation of Midwives (ICM) criteria, and only two were led by midwives. In Nigeria, all infrastructure, curriculum and student- and regulatory-related standards examined were met, but gaps were identified in program staffing and preceptor capacity, and support for faculty and clinical practice sites. In both countries, none of the programs met the average number of clinical practice experiences stipulated in the ICM guidelines due to a lack of teaching hospitals. Students in both countries felt safe on campus but did not feel safe traveling to and from the schools and clinical practice sites.

**Conclusions:**

More investment in midwifery education is needed to ensure international standards for quality education are met. Proactive measures are needed to enhance student safety between school and practice settings in conflict-affected settings. By addressing these gaps, we can strive toward improving midwifery education.

**Supplementary Information:**

The online version contains supplementary material available at 10.1186/s12960-025-00977-6.

## Background

Midwives, educated and regulated to international standards, can provide 87% of essential care needed for women and children [1]. Increases in coverage of interventions delivered by midwives have been shown to result in significant gains in averting maternal and neonatal deaths [2, 3]. For example, a recent modeling study found that scaling up coverage of midwifery services would avert 41% of maternal deaths, 39% of neonatal deaths, and 26% of stillbirths, leading to over two million lives saved by 2035 [2].

In humanitarian settings where provider shortages are often exacerbated by conflict and displacement, midwives play a vital role in the provision of maternal, neonatal, sexual and reproductive health services [3]. Their knowledge, skills, proximity to the community and ability to serve on the frontlines make them valuable assets for delivery of high quality and efficient sexual, reproductive, maternal and newborn health services in conflict and disaster settings [4]. However, to do so, midwives must possess the requisite skills and competencies acquired through good‐quality pre-service education. There is substantial heterogeneity in approaches to, content, duration and quality of midwifery education across countries. Three common approaches to midwifery pre-service education are direct entry (nursing degree is not required), post-nursing qualification (training in midwifery after individual obtains nursing degree), and integrated programs (where individuals receive a dual degree in midwifery and nursing) [4, 5]. This study assesses midwifery pre-service education in two conflict-affected settings in Sub-Saharan Africa: Somalia and Nigeria.

Over the past decade, Somalia has expanded its midwifery education significantly, and currently offers a mix of direct-entry programs, typically lasting 3–4 years, and post-nursing education programs, typically lasting 18 months [6]. Similarly, in Nigeria, post-nursing education programs exist but in recent years direct-entry programs have been introduced, with basic midwifery in the state of Yobe commencing in March 2013 and a community midwifery program commencing in March 2018 [5, 7, 8].

In conflict settings, providing midwifery students with the essential competencies and full range of skills needed to deliver services is increasingly important. However, there is little evidence on the extent to which programs in these settings are delivered with sufficient quality to prepare students for work in facilities and communities affected by conflict. How insecurity, conflict and displacement impact pre-service midwifery education programs is also not fully understood. This study set out to assess the extent to which midwifery pre-service education programs in conflict-affected areas of Nigeria and Somalia meet global and local standards. We assessed evidence-based educational inputs and influencing factors articulated in a validated midwifery education assessment tool that are each directly related to achievement of midwifery students’ competences. Our analysis provides important insights on the state of midwifery education in the two countries and shed light on important gaps that require the attention of stakeholders.

## Methods

### Study setting

#### Nigeria

In Nigeria, research was conducted in Yobe State located in the Northeast Zone of Nigeria. Yobe has been acutely affected by the decade-long conflict around the Boko Haram insurgency, with the majority of people living in security-compromised areas and experiencing high displacement, social and economic suffering, and severe food insecurity [9, 10]. Some health facilities have been attacked, looted, or shut down. There is also a serious shortage of health workers in the country (1.83 health workers per 1000 population) [11]. Challenges with maternal and newborn health (MNH) service delivery include difficult terrain and lack of access and shortage of and poor training of health workers [12]. Overall, Nigeria has a high maternal mortality ratio of 1047 deaths per 100,000 livebirths, but in the Northeast, the situation is even worse with more than 1500 per 100,000 live births [13].

Yobe, was one of the first states in northern Nigeria to receive approval for a 2-year curriculum to train community midwives to serve in primary healthcare facilities [8]. There are currently two midwifery programs in Yobe, a 2-year community midwifery program and a 3-year basic midwifery program (Table 1). The basic midwifery and community midwifery programs train midwives with different scopes. The community midwifery program seeks to prepare a lower cadre of competent and skilled midwifery practitioners who are capable of providing midwifery services to individuals and expectant families primarily in homes and in primary health care centers within communities. In contrast, the basic midwifery program is a more comprehensive program whose graduates are able to handle both normal and complex midwifery cases, including high-risk and emergency care, with clinical rotations spanning primary to tertiary hospitals. In contrast, the community midwifery program focuses on domiciliary care and managing normal cases, teaching graduates to identify and refer complications, with most clinical experience concentrated in primary healthcare centers.

**Table 1:** Study Settings and key indicators

	Nigeria	Somalia
Map of study sites		
Nature of conflict	Insurgency by armed group resulting in large-scale forced migration	Protracted armed conflict and drought resulting in large-scale displacement
Beginning of conflict	2009 [17]	1991 [17]
Maternal Mortality Ratio	1047 deaths per 100,000 live births (2020) [13]	621 maternal deaths per 100,000 live births (2020) [14]
Neonatal Mortality Rate	34 neonatal deaths per 1000 live births (2022)	35 neonatal deaths per 1000 live births (2022) [21]
Stillbirths	42.9 per 1000 births (2015) [19]	28 stillbirths per 1000 births (2021) [18]
Skilled Birth attendance	43% (2018) [13]	32% (2020) [20]
Number of health workers	18.3 per 100,000 population [11]	4 per 10,000 population [15]
Type of midwifery education programs included in the study	Direct-entry community and basic midwifery programs	Direct-entry programs
Duration of midwifery programs	Community diploma: 2 years Basic diploma: 3 years	Diploma: 3 years Bachelor’s: 4 years

#### Somalia

In Somalia, research was conducted in the regions of Banaadir and Galgaduud. Like the rest of the country, the regions have faced challenges due to internal conflicts, political instability, and the presence of armed groups, resulting in widespread displacement, infrastructure loss, and disruption of healthcare. The maternal mortality ratio is 621 maternal deaths per 100,000 live births [14]. Currently there are four health care workers per 10,000 population [15]. Somalia has introduced several reforms to its midwifery programs to address its health workforce shortage. While many private universities began providing nursing and other health sciences programs after the collapse of the central government, skilled midwives remained scarce. In response to this challenge, the Ministry of Health (MoH) of the Somalia Federal Government and the United Nations Population Fund (UNFPA) established four midwifery training institutions nationwide in 2012 [16]. Additionally private universities started providing separate midwifery programs. There are a mix of degrees in Somalia, but for this assessment we only focus on direct-entry programs, which are a 3-year diploma program and a 4-year bachelor’s program (Table 1).

### Research design

We conducted a cross-sectional systematic assessment using an adaptation of the Midwifery Education Rapid Assessment Tool developed by Jhpiego and endorsed by the International Confederation of Midwives (ICM) and UNFPA [21]. The Rapid Assessment Tool includes a set of standards, verification criteria, and a scoring rubric that provide a summative account of “yes” (meets criteria) or “no” (needs priority attention) which were used to identify gaps and bottlenecks in six domains of pre-service education: infrastructure and management, teachers and preceptors, clinical practice sites, curriculum, and students, and influencing factors. The research team adapted the tool to each of the two contexts by adding questions relevant to national standards and incorporating questions specific to the impact of conflict on access to education in the two settings, including challenges getting to school, feelings of safety at clinical practice sites and on school premises Questions that are only applicable to country-wide/national indicators which require an assessment of all midwifery programs were removed. The tools (Supplementary File A) were pilot tested in the Kano Midwifery School in Nigeria and Abrar University in Somalia, which were not in the sample of schools selected.

### Study site selection

The two public midwifery programs in Yobe were both invited to participate. In Somalia, of 16 schools, seven schools were purposively selected and agreed to participate in the study, six in Banaadir and one in Galgaduud. Our criteria included type of degree program offered (3-year diploma vs 4-year bachelor programs), year the school was established, and type of school (private vs public). We wished to represent older and more newly established schools as well both public and private 3- and 4-year programs so we purposively selected the schools based on these criteria.

### Data collection

Data collection took place in November 2022 in Nigeria and over the period December 2022 to February 2023 in Somalia. Information was collected through structured interviews, based on the adapted Rapid Assessment Tool, with midwifery education program faculty and students during site visits to schools and associated clinical practice sites. Sample size for each category of participants was determined based on guidance set out by the Rapid Assessment Tool. In each school, data collectors interviewed a program director or a designate if the program director was not available during the visit, two teachers per program, 10 students per program, and one to two clinical preceptors per practice site per school (in schools that had several practice sites, at least two facilities were visited and at least one preceptor was interviewed). We used preset eligibility criteria to select participants. Program directors were eligible for inclusion if they were either appointed or temporarily acting as head of the program. Teachers were eligible if they were in the school for at least one academic year in a full-time position. Students were eligible if they were in their second year of the program and preceptors had to have been working in their current role for at least 1 year. Participants were randomly selected by the dean of the school from those who expressed interest and willingness to participate in the study after an announcement by the dean.

In addition, using structured surveys that reflect the Rapid Assessment Tool standards, we interviewed key stakeholders including MoH officials, community leaders, school provosts, representatives of health professional associations and UN agencies (Table 2). A total of five male and female data collectors were trained in Nigeria and two male and female data collectors in Somalia. All data collectors were trained in human subject research and research tools over a 2-day training and subsequently had the opportunity to participate in the pilot-testing. All interviews were administered by the data collectors using KoBo Toolbox.

**Table 2:** Participants interviewed in each study setting

Type of participant	Nigeria (2 schools)	Somalia (7 schools)
	N	N
Program directors	2	7
Teachers	4	14
Students	20	70
Clinical preceptors	4	10
Stakeholders	9	5
Total	39	106

### Data analysis

All data, including names of schools and interviewed individuals, was anonymized, and then analyzed on Excel and Stata 15. The teams conducted descriptive analysis, including calculation of means and percentages, and assigned scores based on the scoring rubric and guidance outlined in the Rapid Assessment Tool. Three of the standards in Somalia were not scored because the team was not able to collect the complete information needed for scoring. Validation activities took place in both countries in September 2023. Stakeholders with expert knowledge of midwifery education in each country were invited to a 1-day workshop where the team presented results and solicited feedback and reactions. This ensured that the findings were sound and trustworthy and allowed decision-makers to participate in the formulation of recommendations and policy solutions based on the results.

### IRB approval

The study received Institutional Review Board (IRB) approval from the Johns Hopkins School of Public Health IRB (21640), Somali Research and Development Institute’s Research Board (SORDI-EA02 46), and the Yobe State Health Research Ethics Committee (MOH/GEN/747).

## Results

In Nigeria, each school met between 17 to 18 standards respectively out of 22 across assessment domains (77.3%–81.8%). In contrast, in Somalia, schools met between 6 to 10 standards out of the 19 standards for which data was available (31.6%–52.6%). Below, we summarize the findings from the assessment. Table 3 summarizes school-specific findings across domains.

**Table 3:** Summary of scoring per domain

Indicator	Somalia							Nigeria	
	A	B	C	D	E	F	G	A	B
I. Curriculum									
The curriculum is competency-based	**Yes**	**Yes**	**Yes**	**Yes**	**Yes**	**Yes**	**Yes**	**Yes**	**Yes**
The curriculum has been reviewed and updated within the past 5 years	**Yes**	No	**Yes**	**Yes**	**Yes**	No	**Yes**	**Yes**	**Yes**
The curriculum is aligned with national health priorities, and has been endorsed by the ministry of health and relevant bodies	NA	NA	NA	NA	NA	NA	NA	**Yes**	**Yes**
II. Teachers and preceptors									
The school has sufficient midwives and appropriate non-midwives that are needed to educate existing students in the academic/theory components of the curriculum	No	No	No	No	No	**Yes**	No	No	No
Teachers have completed a course preparing them for their teaching role	**Yes**	**Yes**	**Yes**	**Yes**	**Yes**	**Yes**	**Yes**	No	**Yes**
Teachers have acquired and maintain their clinical competency	No	No	No	No	No	No	No	**Yes**	**Yes**
Teachers have the resources that they need to be effective	No	No	No	No	No	No	No	**Yes**	**Yes**
Teachers receive salary equal to or greater than midwives in clinical practice	NA	NA	NA	NA	NA	NA	NA	**Yes**	**Yes**
The education system has clinicians prepared for the role of clinical preceptor (clinical teacher)	No	No	No	No	No	No	No	No	No
The education system has clinicians supported in the role of clinical preceptor (clinical teacher)	NA	NA	NA	NA	NA	NA	NA	No	No
III. Infrastructure									
The midwifery program or school being assessed is led by a midwife with appropriate clinical, administrative, academic and leadership experience	No	No	No	No	No	**Yes**	**Yes**	**Yes**	Yes
The school being assessed has sufficient space needed to facilitate theoretical (classroom) learning needs of students	**Yes**	**Yes**	No	No	No	No	No	**Yes**	**Yes**
The school has the textbooks and journals or library Internet access to journals, and other library resources needed for existing students	No	No	No	No	**Yes**	**Yes**	No	**Yes**	**Yes**
The school has a functional clinical skills lab needed for practice and simulation	No	No	No	No	No	No	No	**Yes**	**Yes**
The school has a computer lab with sufficient functional computers, and appropriately skilled teaching/support staff	No	No	No	Yes	No	No	No	**Yes**	**Yes**
IV. Students									
The school is located in communities accessible to targeted students (commutable from residence to school on a daily basis)	**Yes**	**Yes**	No	**Yes**	**Yes**	**Yes**	**Yes**	**Yes**	**Yes**
The majority of students enrolled are enthusiastic about entering the midwifery profession	**Yes**	**Yes**	**Yes**	**Yes**	**Yes**	**Yes**	**Yes**	**Yes**	**Yes**
The country and/or school has student selection criteria that account for anticipated deployment and retention	No	No	No	No	No	Yes	Yes	Yes	Yes
V. Clinical practice sites									
The school has sufficient clinical sites needed to prepare students to competency in accord with ICM guidelines	No	No	No	No	No	No	No	No	No
The school has clinical practice sites that are accessible to students and teachers (commutable from school to clinical facility in accord with the schedule of clinical experiences)	**Yes**	**Yes**	**Yes**	No	**Yes**	**Yes**	**Yes**	**Yes**	**Yes**
The clinical practice site has sufficient medical supplies and other resources needed to train students to competency	No	No	**Yes**	No	**Yes**	No	**Yes**	**Yes**	**Yes**
The clinical practice site models practice that is consistent with evidence-based best practices	No	No	No	No	No	**Yes**	**Yes**	**Yes**	**Yes**

*NA* Not assessed owing to inability to obtain needed data for scoring of the indicator

### Curriculum

Three standards for midwifery pre-service education curricula were assessed, namely that the curriculum (1) is competency based, (2) has been reviewed and updated within the past 5 years, and (3) is aligned with national health priorities endorsed by the MoH and relevant bodies.

All three of these standards were met by both programs assessed in Yobe State, Nigeria. The curriculum in Yobe State is the standardized curriculum approved by the Nursing and Midwifery Council of Nigeria (NMCN) which is the national accrediting body for nursing and midwifery programs. Both curricula are competency-based and were reviewed and updated within the past 5 years.

In Somalia, findings were varied. All assessed schools use different teaching curricula and assessment methods, underscoring important inconsistencies. The curricula of both public schools are reviewed and updated every 5 years; however, they had different review processes and durations. Two out of the four private schools did not implement a review or update process for curricula. All seven schools fulfilled the competency-based requirement, combining simulation with clinical practice opportunities and varied teaching approaches.

### Teachers and preceptors

Seven standards for midwifery pre-service education faculty (teachers and preceptors) were assessed which covered the following criteria: availability of teachers, their preparation to teach, access to teaching resources; remuneration; acquisition and maintenance of competency.

Teacher to student ratios in Nigeria, ranged from 1:40 to 1:83 in the basic program and 1:33 in all three observed courses in the community program. Both programs in Nigeria met the criteria for availability of qualified teachers for all courses observed, but they did not meet the overall standard because of high student-to-teacher ratio. In Somalia, there was great heterogeneity across the seven schools, ranging from 1:25 to 1:59. Between the two countries, only one school in Somalia met the required 30 students per teacher ratio. Six out of the seven schools in Somalia fell short of the International Confederation of Midwives (ICM) standard for sufficient number of teaching faculty, i.e., number of midwives and suitable non-midwives on staff needed to educate students in the academic/theory components of the curriculum.

In Nigeria, only one of the two programs met the teaching preparation requirement, with teachers reporting completion of the preparation courses in the community midwifery program but not the basic program. Both teachers however met the competency standard, which requires teachers to have acquired clinical practice prior to transferring to the role of academic teacher and/or to have practiced in a clinical setting within the past year. In contrast, all teachers in Somalia completed the required teacher preparation courses in all the schools but none met the competency standard.

In terms of resources for effective teaching, at least two midwifery teacher offices were assessed in each school to identify whether teachers have the necessary resources. Our findings revealed that, participating schools in Nigeria programs have sufficient resources. However, none of the schools in Somalia met this standard. Seventy per cent of the schools in Somalia lacked an office with a desk for every teacher, but most of them had access to internet, working electricity, toilets, running water, and office supplies.

In Nigeria, the criteria for remuneration were met in both programs because the majority of midwifery educators are employed through the MoH within the Consolidated Health Salary Structure (CONHESS) for healthcare workers. Hence, they earn equivalent salaries as their counterparts in the clinical practice areas. However, if a midwifery educator is employed directly into the academic institution on the Consolidated Polytechnics and Colleges of Education Academic Staff Salary Structure (CONPCASS), he/she may start at a lower salary level when compared to the healthcare worker salary structure (CONHESS). In Somalia, we were unable to get complete information on teachers’ salaries and thus this standard was not scored.

For preceptor availability and student ratio, none of the programs in Somalia or Nigeria had enough preceptors available for students on clinical rotations, with preceptor-to-student ratios falling short of the ICM recommended guidance of 1:4. In terms of preceptor training and preparation, none of the preceptors received any formal preparatory training for their roles. As such, this standard was neither met by the basic nor community programs. In Somalia, this standard was not evaluated because the team was not able to obtain all the needed data required for scoring.

### Infrastructure and management

Five educational standards assessed quality of infrastructure and management, capturing issues such as the physical environment in the school, including availability of desks for every student, good ventilation, light, and other aspects of the learning environment.

All five standards under this domain were met in Nigeria but most were not met in Somalia. Both schools in Nigeria had a leader who is qualified as a midwife with at least 2 years of teaching experience in the nursing school as well as clinical experience. In contrast, in Somalia, only the two public midwifery schools were led by qualified midwives whereas, all the private midwifery schools assessed were led by non-midwives. All school directors had teaching experience for more than 2 years and five out of seven directors had prior experience in administration.

In Nigeria, the country-specific maximum capacity was 100 students per classroom, and class size ranged between 57 and 100. In Somalia, all classrooms were appropriately equipped for teaching purposes. All students had individual desks within elbow reach, and the classrooms were well-ventilated and had enough light. The number of students per classroom ranged between 30 and 50, with all schools exceeding the country-specific maximum capacity of 30 students per classroom except two private schools that adhered to the 30-student benchmark.

In Nigeria, both schools had a library with the recommended textbooks, internet access to electronic books, and a designated head of library. Schools also had functional skills and computer labs with the necessary equipment. In Somalia, six out of seven schools had a library, but only two schools had the recommended textbooks in their library. Around 70% of the schools assessed had internet access to electronic texts and a designated head for the library. Half of the schools did not have a budget allocated for buying new resources. Six out of seven schools had functional skill labs with most of the equipment present. However, an IUD insertion kit and a cervical insertion model were missing from most of the schools. Some universities had skilled lab managers, while others required each teacher to be responsible for their subject and provide practice to the student. Only 60% of schools had schedules for student use. Most of the schools (6 out of 7) did not have a computer lab with sufficient functional computers, for the students enrolled in the midwifery program.

### Students

In Nigeria, students across both schools reported no issues with accessibility as most students either lived in the catchment areas of the schools or in campus residence facilities. Students were given extra consideration for admission based on their region of residence, status as a member of an underserved or minority group and intention to practice in an underserved area after graduation. In Somalia, all but one of the schools were in locations accessible to students, with one school located on the outskirts of Mogadishu, posing difficulty for students to travel to the school. Still, students reported problems getting to campus due to traffic jams and road blockages due to insecurity. In Somalia, none of the private schools gave special consideration for students based on region of residence and marginalized group. Public schools were the exception as they gave special consideration based on state of residence but not those belonging to marginalized groups. Most students admitted to the schools in both countries were enthusiastic about entering the profession.

### Clinical practice sites

Four standards assessed the availability of clinical sites, their accessibility to students and teachers, the availability of medical supplies and other resources needed for training students, and the extent to which practice sites adhere to evidence-based/best practice.

In Nigeria, both programs had a sufficient number of clinical sites where students are placed for practical experience, but neither program met the criteria for average number of clinical practice experiences as stipulated in the ICM guidelines. The indicator assessed clinical practice experiences across six areas: new antenatal visits, repeat antenatal visits, labor and birth, newborn examinations, primary care/family planning, and postpartum visits. The basic midwifery program did not meet the average number criteria in any area, but the community program fell short in the three areas of labor and birth, newborn examinations, and primary care/family planning. Both schools in Nigeria had accessible clinical practice sites. In Nigeria both programs met all criteria for availability of medical supplies as well as the required standards for evidence-based practice.

In Somalia, four out of the seven schools had sufficient clinical sites where students are placed for practical experience, but none of them met the average number of clinical practice experiences. All but one of the schools in Somalia had clinical practice sites that were accessible for teachers and students. In terms of availability of medical supplies, only three out of seven schools in Somalia had the sufficient medical supplies needed to train students. While all wards were under the supervision of qualified midwife 24 h, only two of the sites had clinical practice guidelines available.

### Influencing factors

Indicators under this domain assessed the availability of quality standards, whether a functional accreditation system was in place in the country and whether there was a mechanism for evaluating the competency of graduates prior to deployment.

With respect to regulation, the stakeholders surveyed in Nigeria agreed that there is a regulatory body—the Nursing and Midwifery Council of Nigeria—which reviews and accredits all midwifery institutions. They also reported that Nigeria has a national mechanism for assessing the competency and performance of students before issuing their license. In Somalia, stakeholders disagreed about whether a regulatory body is in fact functional. Two out of five stakeholders stated that the country has a regulatory body, while three were not aware of one. All interviewees in Somalia agreed that midwives do not have a mechanism for providing input or a process for internal review of the program. The stakeholders in Somalia also agreed that the country does not have a licensing exam before deploying students in the health system.

### Safety and security

In Nigeria, 95% of students reported feeling safe on campus and at clinical practice sites. None reported challenges with tardiness or absence due to insecurity. Similarly, all teachers who were interviewed reported feeling safe on campus and did not face challenges getting to campus. However, 50% of preceptors reported concerns over the safety and security of their students while travelling to the facility.

In Somalia, over a tenth of students and 42% of teachers reported issues with getting to the school due to insecurity. There was also general concern from educators about the safety and security of their students while travelling to the facility. Once in school however, most felt safe, with 99% of students reporting feeling safe on campus and 94% reporting feeling safe at clinical practice sites.

## Discussion

Midwifery pre-service education programs in Yobe State, Nigeria met all infrastructure, curriculum and student- and regulatory-related standards examined using the Rapid Assessment Tool. Some standards related to faculty and clinical practice opportunities were met, however gaps were identified in program staffing and preceptor capacity and support for both faculty and clinical practice sites. The extent to which participating pre-service education programs in Banaadir and Galgaduud, Somalia met standards examined using the Rapid Assessment findings were more variable, with the most notable gaps identified under the teaching and infrastructure domains.

Differences in findings across assessment sites in Nigeria and Somalia are not surprising considering the differences in the maturity of midwifery education and workforce development initiatives and regulation in the two countries. Initial field testing of the Rapid Assessment Tool in Myanmar in 2015, and subsequent use in Afghanistan in 2017, also revealed notable variation in the qualifications of teachers and preceptors, availability of recommended resources, and lack of adequate clinical practice opportunities [21, 22]. Gaps identified in both countries were also consistent with those highlighted in a recent scoping review examining evidence related to pre-service midwifery education in sub-Saharan Africa, suggesting that challenges are not unique to contexts affected by conflict and humanitarian crises [23]. However, due to the fragility of human resources for health in conflict-affected settings, additional consideration may be needed in designing strategies to facilitate and sustain improvements.

Continued and increased investment in midwifery workforce development in conflict-affected settings is highlighted as a critical area for strengthening midwifery education for universal health coverage in the UNFPA, UNICEF, WHO, ICM Framework for Action published in 2019 [24, 25]. Rapid assessment findings in both Nigeria and Somalia highlight a need for identifying and advancing innovative strategies to provide supervised clinical practice experience that meets global standards for competency-based midwifery education. To facilitate this will require investments in training, resources and continuing professional development for midwifery educators, as well as investments in increasing the number of health facilities with capacity to offer students supervised practice experience and preceptors to provide direct oversight and mentorship.

Safety and security of both faculty and students takes on even greater importance in conflict-affected settings, particularly where armed conflict has included attacks on health and education. A limited literature describes interventions or measures to mitigate disruptions to education, with most studies focusing on documenting impacts on health professionals training and education. A recent scoping review of the impacts of the Syria conflict on education and training for health workers included interventions like tele-training and online teaching, community-based learning and peer-led trainings as innovations to cope with the disruptions to education caused by armed conflict [26]. In both Nigeria and Somalia study sites, students generally felt safe within school premises but described risks and challenges faced in commuting to and from schools and clinical practice sites. Testing effectiveness of interventions like housing benefits or subsidies can help address student safety in conflict settings.

Although beyond the scope of this rapid assessment, an additional question for further study is the extent to which students are being trained on minimum standards for humanitarian service delivery or to address context-specific health issues such as management of co-morbidities associated with severe undernutrition, infectious disease outbreaks, measures to address psychosocial distress among patients characteristic of many conflict settings, as well as self-care measures [27, 28]. Both pre-service preparations, and either routine or ‘just-in-time’ in-service training strategies are important for upscaling quality midwifery care in humanitarian settings [3, 29].

Our study should be considered in light of certain limitations. Firstly, we relied on an established assessment tool, which may not fully account for context-specific features of midwifery programs in the study sites. We strove to adapt the tool to the study contexts, and removed or added questions to ensure that the tool was suitable for the two settings and that global standards were harmonized with national standards when discrepancies arose. We were additionally constrained by the data that was available and were thus unable to assess some standards. Moreover, we did not conduct a national assessment of all midwifery schools in Nigeria and Somalia so our findings are not generalizable at the national level. We also did not randomly select participants in each school and relied on the school director to do that. Nonetheless, we included all programs in Galgaduud, a substantial number of programs in Banaadir, and all programs in Yobe state, so we are comfortable drawing conclusions on the quality of midwifery pre-service education in these areas.

Despite limitations, this rapid assessment provides new evidence that has potential to influence midwifery education policy and practice in study locations, and globally. In Nigeria, study findings raise a number of practical issues for consideration as the Government of Nigeria implements ongoing collegiate nursing and midwifery education reforms and expands its community midwifery and nursing programs to address the shortage of skilled health workers in rural communities. With these reforms currently underway, there is an opportunity to reassess teacher and clinical preceptor availability and preparedness which were identified as two main weaknesses. In Somalia, findings highlight a number of current gaps in midwifery education as well as the need for investment in regulation, one of the three pillars of the ICM’s three-pillar approach to strengthening midwifery [30]. Other recommendations include the need to revise the admission processes across schools to ensure that applicants who belong to underserved communities or minority groups are prioritized in enrollment. Additionally, investments in midwifery infrastructure and built environment are critically needed. The country is currently revising its National Midwifery Strategy, and the study recommendations have been considered in the ongoing strategy consultation. Moreover, dissemination of assessment findings among participants and stakeholders in study areas is helping raise awareness of national and international standards for both midwifery education and service delivery, providing tools for midwifery service providers, educators and stakeholders to advocate for greater investment and accountability to strengthen systems that are critical for the health and wellbeing of women and children.

## Conclusions

Midwifery pre-service education is a foundation for essential maternal and newborn health service delivery in all settings, including those affected by armed conflict and insecurity. This rapid assessment of programs in two distinct settings, Yobe State in Nigeria, and Banaadir and Galgaduud in Somalia, highlights the importance of standardizing competency-based curricula, regularly assessing the readiness of schools to prepare qualified clinicians, and ensuring adequate systems and supports are in place, specifically in conflict-affected settings, to facilitate learning in a safe and secure environment in a health system under stress.

## Supplementary Information

Supplementary Material 1.

## Data Availability

The de-identified dataset will be posted on Figshare (link to be added).

## Abbreviations

MOH: Ministry of Health
ICM: International Confederation of Midwives
MNH: Maternal and newborn health
UNFPA: United Nations Population Fund
