# Supplementary material for: Rapid assessment of pre-service midwifery education in conflict settings: findings from a cross-sectional study in Nigeria and Somalia
Source: Hum Resour Health. 2025 Feb 3;23:6. doi: 10.1186/s12960-025-00977-6 (PMC11792377; doi:10.1186/s12960-025-00977-6)
Supplement: Supplementary file 1 — Supplementary Material 1. [file 12960_2025_977_MOESM1_ESM.pdf]

Tool A: Interview with Program Director (or designate)

**Country**	` ◻ Nigeria	◻ Somalia
**Location**	`◻ Yobe	`◻ Mogadishu<br>◻ Galguduud
**Institutional affiliation**	◻ Government	◻ Private
**Name of school**		
**Type of program**		
**Interviewee**	◻ Program Director	◻ Designate

**Data collector name**	
**Date of interview**	

**Interview start time**	

INTRODUCTION (Nigeria)
Good Morning/Afternoon/Evening: My name is Mr. /Mrs. /Ms. /Dr……………………………………… I am a Research Assistant with the Institute of Human Virology, Nigeria working as part of a project called EQUAL which seeks to improve maternal and newborn health outcomes. We are currently conducting a research study on Midwifery Education in Conflict-Affected Areas of Nigeria. The study seeks to provide deeper understanding and actionable insights into issues influencing midwives' ability to deliver quality MNH services findings. Findings will help inform programmatic recommendations that will potentially benefit midwifery students and educators and result in a stronger local health workforce in the future. This part of the study will assess the extent to which midwifery pre-service education programs in EQUAL study locations meet national and global (International Confederation of Midwives) standards. It will also explore how conflict affects pre-service midwifery education in EQUAL study locations.

This study will involve asking you some questions about your impressions and experiences with this midwifery program. Participation is completely voluntary – you can choose not to participate, and even if you agree to participate, you may stop at any time. Any information we collect is confidential. We will not record your names and no personal individual information will be shared with anyone.
There will be no direct benefit to you from participating in this study. We are asking for your help to collect information to improve midwifery education in Nigeria.

If you wish to participate in the study, I will now proceed to ask you a series of questions.
May I continue?

If "no" → Thank you for your time and have a nice day.

In case you need more information about the survey, you may contact the person listed on this card [PROVIDE CARD OR PAPER WITH STUDY CONTACT].

INTRODUCTION (Somalia)
Good Morning/Afternoon/Evening: My name is Mr. /Mrs. /Ms. /Dr……………………………………… I work with the Somali Research and Development Institute (SORDI) working as part of a project called EQUAL which seeks to improve maternal and newborn health outcomes. We are currently conducting a research study on Midwifery Education in Conflict-Affected Areas of Somalia. The study seeks to provide deeper understanding and actionable insights into issues influencing midwives' ability to deliver quality MNH services findings. Findings will help inform programmatic recommendations that will potentially benefit midwifery students and educators and result in a stronger local health workforce in the future. This part of the study will assess the extent to which midwifery pre-service education programs in EQUAL study locations meet national and global (International Confederation of Midwives) standards. It will also explore how conflict affects pre-service midwifery education in EQUAL study locations.
This study will involve asking you some questions about your impressions and experiences with this midwifery program. Participation is completely voluntary – you can choose not to participate, and even if you agree to participate, you may stop at any time. Any information we collect is confidential. We will not record your names and no personal individual information will be shared with anyone.

There will be no direct benefit to you from participating in this study. We are asking for your help to collect information to improve midwifery education in Somalia.

If you wish to participate in the study, I will now proceed to ask you a series of questions.
May I continue?

If "no" → Thank you for your time and have a nice day.

In case you need more information about the survey, you may contact the person listed on this card [PROVIDE CARD OR PAPER WITH STUDY CONTACT].

Basic School Information

1.1	Year of program establishment	
1.2	Number of cohorts enrolled to date	
1.3	Number of cohorts graduated to date	
1,4	Average number of students per cohort	
1.5	Number of students currently enrolled	Cohort 1 ______<br>Cohort 2 ______<br>Cohort 3 ______

1. Information about Program Director

Ask the Director to tell you about their professional background. Complete the following questions.

2.1	Gender	□ Male<br>□ Female
2.2	Qualification	□ Midwife<br>□ Not Midwife<br>If not Midwife, specify qualification__________<br>*If not a midwife, skip to question 2.5*
2.3	Highest level of midwifery qualification	□ Diploma Midwife<br>□ Bachelors degree<br>□ Masters degree<br>□ PhD<br>□ Other<br>If other, specify qualification___________
2.4	Years of clinical experience in midwifery practice	
2.5	Years of teaching experience	
2.6	Prior experience in administration of a higher education program	`□ Yes<br>□ No
2.7	Prior experience in management of a clinical unit at district hospital or higher level facility	□ Yes<br>□ No
2.8	Prior experience supervising or in charge of work of 5 or more people	□ Yes<br>□ No

2. Information about Students

3.1	**Does the school manage its own admissions processes?**	□ Yes, admissions are managed by school<br>□ No, admissions are managed by FMoH or FMoHE<br>□ No, admissions are managed by other entity<br>If managed by other entity, please explain__________
3.2	**Are all available student slots filled for the current academic year?**	`□ Yes<br>□ No
3.3	**What are minimum entry requirements?**	Education level: ______<br>Other (explain) _______
3.4	**Are student applicants given extra consideration for admission based on their residence location** (urban, rural, conflict-affected location)**?**	`□ Yes<br>□ No<br>If Yes, please explain:_______________
3.5	**Are student applicants given extra consideration for admission based on their status as a member of an underserved or minority population group?**	`□ Yes<br>□ No<br>If Yes, please explain:_______________
3.6	**Are students given extra consideration for admission based on their stated intention to practice in an underserved area following graduation?**	`□ Yes<br>□ No<br>If Yes, please explain:_______________
3.7	**Number of qualified applicants this year**	
3.8	**Was there a wait list for enrollment for this academic year?**	`□ Yes<br>□ No
3.9	**Number of students on wait list?**	
3.10	**Do most students live within the area surrounding the school** (within distance accessible by public or private transportation)**?**	`□ Yes<br>□ No
3.11	**Are accommodations (residence facilities) available for students who live at a distance?**	`□ Yes<br>□ No
3.12	**Do you face challenges with**	`□ Yes<br>□ No

	**student tardiness or absences due to transportation difficulties?**	If Yes, please explain:_______________
**3.13**	**Do you face challenges with tardiness or absences due to insecurity issues?**	`□ Yes<br>□ No<br>If Yes, please explain:_______________

3. Information about School Curriculum

**4.1**	**Are there national standards for midwifery education programs?**	`□ Yes<br>□ No
**4.2**	**Do you have a mechanism for conducting internal program reviews?**	`□ Yes<br>□ No<br>If yes, how often are reviews conducted?___________
**4.3**	**Do you have a standard curriculum for midwifery education?**	`□ Yes<br>□ No<br><br>*If no, skip to question 5.1*
**4.4**	**Year curriculum was most recently updated**	
**4.5**	**Who developed/issued the curriculum?**	□ Federal Ministry of Health and/or Higher Education<br>□ UNFPA<br>□ Curriculum developed specifically for this school<br>□ Other<br>If other, please explain__________________________<br><br>*If other, or curriculum developed specifically for school, ask for an electronic or paper copy of the curriculum.*
**4.6**	**Number of courses in curriculum**	
**4.7**	**Time needed to complete curriculum**	____ Years
**4.8**	**What percentage of the curriculum is spent in a classroom or skills lab?**	_____%
**4.9**	**What percentage of the curriculum is spent in at clinical practice sites?**	_____%
**4.10**	**Are all courses taught by individuals with appropriate academic qualifications?**	`□ Yes<br>□ No

4. Information about Course Instruction

**5.1**	**Total number of course instructors currently employed**	____ Full-time<br>____ Part-time
**5.2**	**Number of course instructors that are qualified midwives**	

**5.3**	**Number of course instructors that are NOT midwives**		
**5.4**	**Have all teachers completed pedagogical / instructional skills** (e.g. teacher training) **courses?**	`□ Yes<br>□ No	
**5.5**	**Do most teachers live within the area surrounding the school** (within distance accessible by public or private transportation)**?**	`□ Yes<br>□ No	
**5.6**	**Are accommodations (residence facilities) available for teachers who live at a distance?**	`□ Yes<br>□ No	
**5.7**	**Do you face challenges with teacher tardiness or absences due to transportation difficulties?**	`□ Yes<br>□ No<br>If Yes, please explain:_______________	
**5.8**	**Do you face challenges with teacher tardiness or absences due to insecurity issues?**	`□ Yes<br>□ No<br>If Yes, please explain:_______________	
**5.9**	**Is there a set salary scale for full-time teachers?**	`□ Yes<br>□ No<br>*If yes, ask for copy or where to request information.*_	
Ask to list through all the teaching staff and their course responsibilities..			
**5.10**	**Teacher #1**	***Courses Taught***	
		General Curriculum	***Midwifery***
	**Teacher #2**		
	**Teacher #3**		
	**Teacher #4**		
	**Teacher #5**		
	**Teacher #6**		
	**Teacher #7**		
	**Teacher #8**		
	**Teacher #9**		
	**Teacher #10**		
Explain that you would like to ask for information about a few specific midwifery courses.			
**5.11**	**Course #1**	***Course Name***	
		Number of teachers	
		Techer	□ Midwife

		qualifications	□ Not midwife<br>If not Midwife, specify qualification__________
		Number of students	
		Required textbook(s)	
**5.12**	**Course #2**	***Course Name***	
		Number of teachers	
		Techer qualifications	□ Midwife<br>□ Not midwife<br>If not Midwife, specify qualification__________
		Number of students	
		Required textbook(s)	
**5.13**	**Course #3**	***Course Name***	
		Number of teachers	
		Techer qualifications	□ Midwife<br>□ Not midwife<br>If not Midwife, specify qualification__________
		Number of students	
		Required textbook(s)	

5. Information about School Infrastructure and Resources

Library

**6.1**	**Does this school have its own library?**	`□ Yes<br>□ No<br><br>*If no, skip to question 6.11*
**6.2**	**When is the library open for student use?** (select all that apply)	□ During the same hours as classes<br>□ Evenings<br>□ Weekends<br>□ Other<br>If other, please explain__________________________
**6.3**	**Who manages the library?**	□ Librarian / Head of Library<br>□ Designated Teacher<br>□ School Director<br>□ Other<br>If other, please explain__________________________

**6.4**	**When are they available in the library?** (check all that apply)	□ During the work-week / official school hours<br>□ Evenings<br>□ Weekends<br>□ On request only<br>□ Other<br>If other, please explain_________________________
Ask to see library		
**6.5**	**Does the library have internet access?**	`□ Yes<br>□ No<br><br>*If no, skip to question X*
**6.6**	**Are textbooks available in the library?**	`□ Yes<br>□ No<br><br>*If no, skip to question X*
**6.7**	**Source of textbooks**	`□ Donated<br>□ Purchased<br>□ Other<br>If other, please explain_________________________<br><br>*If no, skip to question X*
**6.8**	**Number of copies of required text books available**	(see table below)
**6.9**	**Does the library have access to scientific / health professional journals?**	`□ Yes<br>□ No

6.8 Number of copies of required text books available

Nigeria		Somalia	
Book	***Number of copies***	***Book***	***Number of copies***
Foundations of Anatomy and Physiology (Ross, JS & Wilson K)		*Anatomy and Physiology in Health and Illness (Ross, JS & Wilson, K)*	
Foundations of Nursing and First Aid (Ross, JS & Wilson K)		*Fundamentals of Nursing*	
Myles Textbook for Midwifery		*Procedure Manual for Nurses and Midwifery*	
Essentials of Midwifery and Obstetric Nursing (Basavanthappa, BT)		*Pharmacology of Midwifery*	
Manual of Midwifery and Gynecological Nursing		*Comprehensive Textbook of Midwifery and Gynecological Nursing*	

6.10	**Does the library have videos demonstrating clinical skills?**	`□ Yes<br>□ No
6.11	**Is there an annual budget for purchase of new library books and resources?**	`□ Yes<br>□ No

Computer Lab

6.12	**Does this school have a separate computer lab?**	`□ Yes<br>□ No<br>*If no, skip to question 6.18*
6.13	**When is the computer lab open for student use?** (select all that apply)	□ During the same hours as classes<br>□ Evenings<br>□ Weekends<br>□ Other<br>If other, please explain_________________________
Ask to see computer lab		
6.14	**Does the computer lab have internet access?**	`□ Yes<br>□ No
6.15	**Number of functional computers**	
6.16	**Do all computers have Microsoft Office** (Word, Excel, etc) **or similar software?**	`□ Yes<br>□ No
6.17	**Do all computers have a USB flash drive port, DVD player or equivalent means of video viewing?**	`□ Yes<br>□ No
6.18	**Is the computer lab staffed by a support person with sufficient skills to assist student learners?**	`□ Yes<br>□ No

Clinical Skills Lab

6.19	**Does this school have at least one skills lab?**	`□ Yes<br>□ No<br>*If no, skip to question 7.1*
6.20	**When is the skills lab open for student use?** (select all that apply)	□ During the same hours as classes<br>□ Evenings<br>□ Weekends<br>□ Other<br>If other, please explain_________________________
6.21	**Who is responsible for scheduling students and maintaining supplies in skills**	□ Skills Lab Manager<br>□ Designated Teacher<br>□ School Director

	**lab?**	□ Other<br>If other, please explain_________________________
Ask to see skills lab.		
**6.22**	**Is the skills lab organized** (not cluttered)**?**	□ Yes<br>□ No
**6.23**	**Is there a schedule for individual or group use of the skills lab?**	□ Yes<br>□ No
Check for availability of the following		
**6.24**	**Electronically programmed manikin or simulator**	`□ Yes, functioning<br>` □ Yes, but NOT functioning<br>□ No<br>` □ Not applicable
**6.25**	**MamaNatalie**	`□ Yes, functioning<br>` □ Yes, but NOT functioning<br>□ No<br>` □ Not applicable
**6.26**	**NeoNatalie**	` □ Yes, functioning<br>` □ Yes, but NOT functioning<br>□ No<br>` □ Not applicable
**6.27**	**Sterilizer**	`□ Yes, functioning<br>` □ Yes, but NOT functioning<br>□ No<br>` □ Not applicable
**6.28**	**Pelvic models**	`□ Yes,<br>□ No
**6.29**	**Bony pelvis**	`□ Yes,<br>□ No
**6.30**	**Cervical dilation models**	`□ Yes,<br>□ No
**6.31**	**Fetal skill, with landmarks**	`□ Yes,<br>□ No
**6.32**	**Newborn resuscitation model and kit**	`□ Yes,<br>□ No
**6.33**	**IUD insertion kit and cervical insertion model**	`□ Yes,<br>□ No
**6.34**	**Perineum cutting and suturing simulators** (e.g. scissors, sponge blocks and expired sutures)	`□ Yes,<br>□ No
**6.35**	**Vaginal speculum**	` □ Yes,<br>□ No
**6.36**	**Functional blood pressure apparatus**	`□ Yes,<br>□ No
**6.37**	**Functional thermometer**	`□ Yes,<br>□ No
**6.38**	**Running water and soap (for cleaning hands before and**	`□ Yes, functioning<br>` □ Yes, but NOT functioning

	**after using equipment)**	□ No
**6.39**	**Hand sanitizer**	`□ Yes,<br>□ No

6. Observation of classes

Ask to observe classes that are in session. Complete the following checklist for each classroom.

Classroom #1		
**7.1**	**Number of students in class during session**	
**7.2**	**Does each student have an individual desk or workspace**	`□ Yes<br>□ No
**7.3**	**Is there a subjective feeling of "elbow room" for each student**	`□ Yes<br>□ No
**7.4**	**Is there adequate light and ventilation?**	`□ Yes<br>□ No
**7.5**	**Is the classroom appropriately equipped for teaching purposes**	`□ Yes<br>□ No<br>If no, explain________________________________
Classroom #2		
**7.6**	**Number of students in class during session**	
**7.7**	**Does each student have an individual desk or workspace**	`□ Yes<br>□ No
**7.8**	**Is there a subjective feeling of "elbow room" for each student**	`□ Yes<br>□ No
**7.9**	**Is there adequate light and ventilation?**	`□ Yes<br>□ No
**7.10**	**Is the classroom appropriately equipped for teaching purposes**	`□ Yes<br>□ No<br>If no, explain________________________________
Classroom #3		
**7.11**	**Number of students in class during session**	
**7.12**	**Does each student have an individual desk or workspace**	`□ Yes<br>□ No
**7.13**	**Is there a subjective feeling of "elbow room" for each student**	`□ Yes<br>□ No
**7.14**	**Is there adequate light and ventilation?**	`□ Yes<br>□ No
**7.15**	**Is the classroom appropriately equipped for teaching purposes**	`□ Yes<br>□ No<br>If no, explain________________________________

7. Clinical Practice Sites

Ask about clinical practice sites and requirements.

**8.1**	**Are there sufficient clinical sites in which to place students for practical experiences?**	`□ Yes<br>□ No
**8.2**	**Number of clinical practice sites**	___ Health centers<br>___ Hospitals
**8.3**	**Does the school provide transportation to clinical sties for students and teachers?**	` `□ Yes<br>□ No
**8.4**	**Do students or teachers report difficulties in making their way to clinical sties that result in absence or difficulties?**	`□ Yes<br>□ No<br>If Yes, explain___________________________________
**8.5**	**Are there standard policies or guidelines with clinical practice requirements for graduation?**	` `□ Yes<br>□ No<br><br>*If yes, ask for a copy or where to obtain information.*
**8.6**	**Are there set numbers of clinical practice experiences that students must conduct on either own (under supervision)?**	`□ Yes<br>□ No<br><br>If yes, please specify:

Practice area	Number of independent (supervised) experiences required	
New antenatal care visits		□ Not applicable
Repeat antenatal care visits		□ Not applicable
Labor and birth (continuity of care)		□ Not applicable
Newborn examinations		□ Not applicable
Other reproductive health (including family planning)		□ Not applicable
Postpartum/postnatal care (day 1 or 2) visits		□ Not applicable
Postnatal care (week 1, 4-6) visits		□ Not applicable

8. Gender, social Inclusion, and safety

**9.1**	**Does this midwifery program prepare students for gender inequities they may face in the workplace?**	□ Yes<br>□ No<br><br>Please explain:

**9.2**	**Does this midwifery program include training on how to care for women affected by gender based violence?**	◻ Yes<br>◻ No<br><br>Please explain:
**9.3**	**Does this midwifery program include training on how to care for women with disabilities?**	◻ Yes<br>◻ No<br><br>Please explain:
**9.4**	**Does this midwifery program include training on how to be safe at work while serving in areas affected by violent insecurity?**	◻ Yes<br>◻ No<br><br>Please explain:

Thank the Director for their time.

Explain that you would like to complete the following activities and ask for their support in doing so.

☐ Interviews with 2 teachers

☐ Interviews with 10 students

☐ Visits to clinical practice sites (including meeting with preceptors)

**Interview end time**	

Tool B: Interview with Teachers

**Country**	` □ Nigeria	□ Somalia
**Location**	`□ Yobe	`□ Mogadishu<br>□ Galgaduud
**Institutional affiliation**	□ Government	□ Private
**Name of school**		
**Type of program**		

**Data collector name**	
**Date of interview**	

**Interview start time**	

INTRODUCTION (Nigeria)

Good Morning/Afternoon/Evening: My name is Mr. /Mrs. /Ms. /Dr……………………………………… I am a Research Assistant with the Institute of Human Virology, Nigeria working as part of a project called EQUAL which seeks to improve maternal and newborn health outcomes. We are currently conducting a research study on Midwifery Education in Conflict-Affected Areas of Nigeria. The study seeks to provide deeper understanding and actionable insights into issues influencing midwives' ability to deliver quality MNH services findings. Findings will help inform programmatic recommendations that will potentially benefit midwifery students and educators and result in a stronger local health workforce in the future. This part of the study will assess the extent to which midwifery pre-service education programs in EQUAL study locations meet national and global (International Confederation of Midwives) standards. It will also explore how conflict affects pre-service midwifery education in EQUAL study locations.

This study will involve asking you some questions about your impressions and experiences with this midwifery program. Participation is completely voluntary – you can choose not to participate, and even if you agree to participate, you may stop at any time. Any information we collect is confidential. We will not record your names and no personal individual information will be shared with anyone.
There will be no direct benefit to you from participating in this study. We are asking for your help to collect information to improve midwifery education in Nigeria.

If you wish to participate in the study, I will now proceed to ask you a series of questions.
May I continue?

If "no" → Thank you for your time and have a nice day.

In case you need more information about the survey, you may contact the person listed on this card [PROVIDE CARD OR PAPER WITH STUDY CONTACT].

INTRODUCTION (Somalia)

Good Morning/Afternoon/Evening: My name is Mr. /Mrs. /Ms. /Dr……………………………………… I work with the Somali Research and Development Institute (SORDI) working as part of a project called EQUAL which seeks to improve maternal and newborn health outcomes. We are currently conducting a research study on Midwifery Education in Conflict-Affected Areas of Somalia. The study seeks to provide deeper understanding and actionable insights into issues influencing midwives' ability to deliver quality MNH services findings. Findings will help inform programmatic recommendations that will potentially benefit midwifery students and educators and result in a stronger local health workforce in the future. This part of the study will assess the extent to which midwifery pre-service education programs in EQUAL study locations meet national and global (International Confederation of Midwives) standards. It will also explore how conflict affects pre-service midwifery education in EQUAL study locations.
This study will involve asking you some questions about your impressions and experiences with this midwifery program. Participation is completely voluntary – you can choose not to participate, and even if you agree to participate, you may stop at any time. Any information we collect is confidential. We will not record your names and no personal individual information will be shared with anyone.

There will be no direct benefit to you from participating in this study. We are asking for your help to collect information to improve midwifery education in Somalia.

If you wish to participate in the study, I will now proceed to ask you a series of questions.
May I continue?

If "no" → Thank you for your time and have a nice day.

In case you need more information about the survey, you may contact the person listed on this card [PROVIDE CARD OR PAPER WITH STUDY CONTACT].

1. Professional background

Ask the teacher to tell you about their professional background. Complete the following questions.

1.1	**Gender**	□ Male<br>□ Female
1.2	**Qualification**	□ Midwife<br>□ Not Midwife<br>If not Midwife, specify qualification__________<br><br>*If not a midwife, skip to question 1.4*
1.3	**Highest level of midwifery qualification**	□ Diploma Midwife<br>□ Bachelors degree<br>□ Masters degree<br>□ PhD<br>□ Other<br>If other, specify qualification___________
1.4	**Country where qualification was obtained**	□Nigeria/Somalia<br>□Outside of the country
1.5	**Years of teaching experience**	Total years teaching experience: _____<br>Years teaching in this program: _____
1.6	**Years of clinical experience in midwifery practice prior to becoming an academic teacher**	
1.7	**Do you still have clinical practice work** (in either public or private health facility)**?**	`□ Yes<br>□ No<br><br>If yes, which areas of practice do you cover?<br>□ Antenatal care<br>□ Intrapartum care<br>□ Postpartum/postnatal care (within 1-2 days of birth)<br>□ Postnatal care (e.g. 1 week, 4-6 week visits)<br>□ Reproductive health (including family planning)<br>□ Other<br>If other, specify qualification___________
1.8	**When did you last attend a "clinical update" training? What was the topic?**	Topic: ________________________________<br><br>Date: ________________________________<br><br>□ Does not remember<br>□ Has not received any "clinical updates" or in-service training since completing pre-service education

2. Teacher offices

Ask to see teacher offices.

**2.1**	**Is the teacher's office in accessible vicinity of classrooms and skills labs**	`□ Yes<br>□ No
Check for availability of the following		
**2.2**	**Individual desk for each teacher**	`□ Yes<br>□ No
**2.3**	**Functional computer, shared with no more than 1 other teacher**	`□ Yes<br>□ No
**2.4**	**Internet access**	`□ Yes<br>□ No
**2.5**	**Electricity functioning majority of working day**	`□ Yes<br>□ No
**2.6**	**Office supplies**	`□ Yes<br>□ No
**2.7**	**Textbooks relevant to the courses to which the teacher is assigned**	`□ Yes<br>□ No
**2.8**	**Access to toilet and running water**	`□ Yes<br>□ No

3. Professional experiences

Ask the teacher a few final questions about their professional experiences.

**3.1**	**Do you have access to the necessary supplies and equipment for teaching your courses at the school?**	`□ Yes<br>□ No<br><br>If no, explain what is missing or reasons access is lacking:_______________________________
**3.2**	**What teaching/learning methods do you use in your courses?** (select all that apply)	□ lecture with group participation<br>□ project-based learning<br>□ seminar/discussion<br>□ debate<br>□ problem-based learning<br>□ simulated practice (for clinical courses)<br>□ preceptorship (for clinical courses)<br>□ other<br>If other, explain:___________
**3.3**	**How do you compute course grades (based on what type of assessment)?** (select all that apply)	□ quizzes and examinations at several points in time (e.g. midterm and final exams)<br>□ student participation in group work and discussion<br>□ student-led presentations on assigned topics<br>□ various types and methods of quizzes/examinations (e.g. multiple choice test, essay, OSCE stations)<br>□ other<br>If other, explain:___________
**3.4**	**In your opinion, how well**	□ Very well prepared

	**prepared are graduates from this program to work as midwives in this area?**	□ Well prepared<br>□ Prepared<br>□ Only somewhat prepared<br>□ Not prepared
**3.5**	**Do you feel safe and secure when on campus?**	`□ Yes<br>□ No<br>If no, explain challenges faced related to safety and security:___________________________________
**3.6**	**Do you face challenges with tardiness or absences due to insecurity issues?**	`□ Yes<br>□ No<br>If Yes, please explain:_______________
**3.7**	**Is there anything else you would like to share with us about your experiences as a teacher in this midwifery program?**	

Thank the Teacher for their time.

**Interview end time**	

Tool C: Interview with Students

**Country**	` ◻ Nigeria	◻ Somalia
**Location**	`◻ Yobe	`◻ Mogadishu<br>◻ Galgaduud
**Institutional affiliation**	◻ Government	◻ Private
**Name of school**		
**Type of program**		

**Data collector name**	
**Date of interview**	

**Interview start time**	

INTRODUCTION (Nigeria)
Good Morning/Afternoon/Evening: My name is Mr. /Mrs. /Ms. /Dr……………………………………… I am a Research Assistant with the Institute of Human Virology, Nigeria working as part of a project called EQUAL which seeks to improve maternal and newborn health outcomes. We are currently conducting a research study on Midwifery Education in Conflict-Affected Areas of Nigeria. The study seeks to provide deeper understanding and actionable insights into issues influencing midwives' ability to deliver quality MNH services findings. Findings will help inform programmatic recommendations that will potentially benefit midwifery students and educators and result in a stronger local health workforce in the future. This part of the study will assess the extent to which midwifery pre-service education programs in EQUAL study locations meet national and global (International Confederation of Midwives) standards. It will also explore how conflict affects pre-service midwifery education in EQUAL study locations.

This study will involve asking you some questions about your impressions and experiences with this midwifery program. Participation is completely voluntary – you can choose not to participate, and even if you agree to participate, you may stop at any time. Any information we collect is confidential. We will not record your names and no personal individual information will be shared with anyone.
There will be no direct benefit to you from participating in this study. We are asking for your help to collect information to improve midwifery education in Nigeria.

If you wish to participate in the study, I will now proceed to ask you a series of questions.
May I continue?

If "no" → Thank you for your time and have a nice day.

In case you need more information about the survey, you may contact the person listed on this card [PROVIDE CARD OR PAPER WITH STUDY CONTACT].

INTRODUCTION (Somalia)
Good Morning/Afternoon/Evening: My name is Mr. /Mrs. /Ms. /Dr……………………………………… I work with the Somali Research and Development Institute (SORDI) working as part of a project called EQUAL which seeks to improve maternal and newborn health outcomes. We are currently conducting a research study on Midwifery Education in Conflict-Affected Areas of Somalia. The study seeks to provide deeper understanding and actionable insights into issues influencing midwives' ability to deliver quality MNH services findings. Findings will help inform programmatic recommendations that will potentially benefit midwifery students and educators and result in a stronger local health workforce in the future. This part of the study will assess the extent to which midwifery pre-service education programs in EQUAL study locations meet national and global (International Confederation of Midwives) standards. It will also explore how conflict affects pre-service midwifery education in EQUAL study locations.
This study will involve asking you some questions about your impressions and experiences with this midwifery program. Participation is completely voluntary – you can choose not to participate, and even if you agree to participate, you may stop at any time. Any information we collect is confidential. We will not record your names and no personal individual information will be shared with anyone.

There will be no direct benefit to you from participating in this study. We are asking for your help to collect information to improve midwifery education in Somalia.

If you wish to participate in the study, I will now proceed to ask you a series of questions.
May I continue?

If "no" → Thank you for your time and have a nice day.

In case you need more information about the survey, you may contact the person listed on this card [PROVIDE CARD OR PAPER WITH STUDY CONTACT].

1. Personal information

Ask the student to tell you about their status in school and interest in midwifery.

**1.1**	**Gender**	□ Male<br>□ Female
**1.2**	**Academic year enrolled in midwifery program**	
**1.3**	**Did you choose midwifery as a profession or did someone else choose or assign midwifery as your profession?**	`□ Self<br>□ Other
**1.4**	**Is there a profession you would prefer over midwifery?**	`□ Yes<br>□ No<br>If yes, specify___________________
**1.5**	**Do you know where you are likely to work when you graduate?**	□ Yes<br>□ No<br>If yes, specify location and/or facility type:____________
**1.6**	**Do you anticipate that midwifery will be your profession for most of your working life?**	□ Yes<br>□ No

2. Experience of the program

**2.1**	**Do you feel safe and secure on campus?**	`□ Yes<br>□ No
**2.2**	**Do you face challenges with tardiness or absences due to insecurity issues?**	`□ Yes<br>□ No<br>If Yes, please explain:_______________
**2.3**	**Do you feed safe and secure at clinical practice sites?**	`□ Yes<br>□ No
**2.4**	**Do you feel you will be well prepared to work as a midwife?**	`□ Yes<br>□ No
**2.5**	**Is there anything else you would like to share with us about your experiences as a student in this midwifery program?**	

Thank the Student for their time.

**Interview end time**	

Tool D: Interview with Clinical Preceptors

**Country**	` □ Nigeria	□ Somalia
**Location**	`□ Yobe	`□ Mogadishu<br>□ Galgaduud
**Institutional affiliation**	□ Government	□ Private
**Name of school**		
**Type of program**		
**Type of health facility**	□ Primary health center<br>□ Public hospital<br>□ Private clinic<br>□ Private hospital	

**Data collector name**	
**Date of interview**	

**Interview start time**	

INTRODUCTION (Nigeria)

Good Morning/Afternoon/Evening: My name is Mr. /Mrs. /Ms. /Dr………………………………………… I am a Research Assistant with the Institute of Human Virology, Nigeria working as part of a project called EQUAL which seeks to improve maternal and newborn health outcomes. We are currently conducting a research study on Midwifery Education in Conflict-Affected Areas of Nigeria. The study seeks to provide deeper understanding and actionable insights into issues influencing midwives' ability to deliver quality MNH services findings. Findings will help inform programmatic recommendations that will potentially benefit midwifery students and educators and result in a stronger local health workforce in the future. This part of the study will assess the extent to which midwifery pre-service education programs in EQUAL study locations meet national and global (International Confederation of Midwives) standards. It will also explore how conflict affects pre-service midwifery education in EQUAL study locations.

This study will involve asking you some questions about your impressions and experiences with this midwifery program. Participation is completely voluntary – you can choose not to participate, and even if you agree to participate, you may stop at any time. Any information we collect is confidential. We will not record your names and no personal individual information will be shared with anyone.
There will be no direct benefit to you from participating in this study. We are asking for your help to collect information to improve midwifery education in Nigeria.

If you wish to participate in the study, I will now proceed to ask you a series of questions.
May I continue?

If "no" → Thank you for your time and have a nice day.

In case you need more information about the survey, you may contact the person listed on this card [PROVIDE CARD OR PAPER WITH STUDY CONTACT].

INTRODUCTION (Somalia)

Good Morning/Afternoon/Evening: My name is Mr. /Mrs. /Ms. /Dr……………………………………… I work with the Somali Research and Development Institute (SORDI) working as part of a project called EQUAL which seeks to improve maternal and newborn health outcomes. We are currently conducting a research study on Midwifery Education in Conflict-Affected Areas of Somalia. The study seeks to provide deeper understanding and actionable insights into issues influencing midwives' ability to deliver quality MNH services findings. Findings will help inform programmatic recommendations that will potentially benefit midwifery students and educators and result in a stronger local health workforce in the future.
This part of the study will assess the extent to which midwifery pre-service education programs in EQUAL study locations meet national and global (International Confederation of Midwives) standards. It will also explore how conflict affects pre-service midwifery education in EQUAL study locations.
This study will involve asking you some questions about your impressions and experiences with this midwifery program. Participation is completely voluntary – you can choose not to participate, and even if you agree to participate, you may stop at any time. Any information we collect is confidential. We will not record your names and no personal individual information will be shared with anyone.

There will be no direct benefit to you from participating in this study. We are asking for your help to collect information to improve midwifery education in Somalia.

If you wish to participate in the study, I will now proceed to ask you a series of questions.
May I continue?

If "no" → Thank you for your time and have a nice day.

In case you need more information about the survey, you may contact the person listed on this card [PROVIDE CARD OR PAPER WITH STUDY CONTACT].

1. Professional background

Ask the preceptor to tell you about their professional background. Complete the following questions.

**1.1**	**Gender**	□ Male<br>□ Female
**1.2**	**Qualification**	□ Midwife<br>□ Not Midwife<br>If not Midwife, specify qualification__________<br><br>*If not a midwife, skip to question 1.4*
**1.3**	**Highest level of midwifery qualification**	□ Diploma Midwife<br>□ Bachelors degree<br>□ Masters degree<br>□ PhD<br>□ Other<br>If other, specify qualification___________
**1.4**	**Country where qualification was obtained**	□Nigeria/Somalia<br>□Outside of the country
**1.5**	**Years of clinical experience in midwifery practice**	
**1.6**	**When did you last attend a "clinical update" training? What was the topic?**	Topic: ________________________________<br><br>Date: ________________________________<br><br>□ Does not remember<br>□ Has not received any "clinical updates" or in-service training since completing pre-service education

2. Information about clinical practice site

Ask the preceptor to tell you about their health facility.

**2.1**	**How many midwives/nurses work in the maternity area on each shift?**	___ Day shift<br>___ Night shift
**2.2**	**How many schools use this facility as a clinical practice site?**	
**2.3**	**Are there set numbers of clinical practice experiences that students must conduct on either own (under supervision) prior to graduation?**	`□ Yes<br>□ No<br><br>If yes, please specify:

**Practice area**	**Number of independent (supervised) experiences required**	
New antenatal care visits		□ Not applicable
Repeat antenatal		□ Not applicable

		care visits		
		Labor and birth (continuity of care)		□ Not applicable
		Newborn examinations		□ Not applicable
		Other reproductive health (including family planning)		□ Not applicable
		Postpartum/postnatal care (day 1 or 2) visits		□ Not applicable
		Postnatal care (week 1, 4-6) visits		□ Not applicable
**2.4**	**On average, how many health professional students are placed in the health facility on one shift (from all institutions)?**			
**2.5**	**Is there a qualified preceptor available on the unit on each shift to which a midwifery student is assigned?**	`□ Yes<br>□ No		
**2.6**	**Do students or teachers report difficulties in making their way to clinical sties that result in absence or difficulties?**	`□ Yes<br>□ No<br>If Yes, explain________________________________		
**2.7**	**Do you have any concerns about student safety and security while traveling to the facility?**	`□ Yes<br>□ No<br>If Yes, explain________________________________		
**2.8**	**Do you receive any adjustment to your workload when students are on site?**	`□ Yes<br>□ No<br>If Yes, explain________________________________		
**2.9**	**Do you think students completing rotations at this facility will be well prepared to work as midwives after graduation?**	`□ Yes<br>□ No<br>If Yes, explain________________________________		
**2.10**	**Is there anything else you would like to share with us about your experiences as a preceptor for midwifery students?**			

3. Site observation

Ask the preceptor to show you each of the following areas and conduct an inventory of supplies in the unit.

Antenatal care

**3.1**	**Functional blood pressure apparatus**	□ Yes<br>□ No<br>If yes, indicate number available: ___
**3.2**	**Fetal stethoscope** (any combination of Pinard, fetoscope, Doppler)	□ Yes<br>□ No<br><br>If yes, indicate number available: ___
Delivery room		
**3.3**	**How many women are currently in labor beds or awaiting admission to labor?**	
**3.4**	**Gloves**	□ Yes<br>□ No<br>If yes, indicate number available: ___
**3.5**	**Uterotonic**	□ Yes<br>□ No<br>If yes, indicate number available: ___
**3.6**	**IV solution and giving set**	□ Yes<br>□ No<br>If yes, indicate number available: ___
**3.7**	**Sterile birth kit**	□ Yes<br>□ No<br>If yes, indicate number available: ___
**3.8**	**Decontamination solution**	`□ Yes<br>□ No
**3.9**	**Newborn resuscitation bag and mask (size 0 or 1)**	`□ Yes<br>□ No

4. Logbook review

Ask to see the register book or monthly reports. Complete the following information

**4.1**	**How many births occurred in the last month?**	
**4.2**	**How many new antenatal client visits in the last month?**	
**4.3**	**How many return antenatal client visits in the last month?**	
**4.4**	**How many postnatal care visits in the last month?**	
**4.5**	**How many family planning client visits in the last month?**	

Thank the preceptor for their time.

**Interview end time**	

Tool E: Interview with Midwifery Education Stakeholders

**Country**	` □ Nigeria	□ Somalia
**Location**	`□ Yobe	`□ Mogadishu<br>□ Galgaduud
**Type of Stakeholder**	□ FMoH/SMoH official<br>□ UN agency<br>□ Health professional association<br>□ Local government official<br>□ Community leader<br>□ Other (specify)___________________	
**Gender**	`□ Male<br>□ Female	

**Data collector name**	
**Date of interview**	

**Interview start time**	

INTRODUCTION (Nigeria)

Good Morning/Afternoon/Evening: My name is Mr. /Mrs. /Ms. /Dr……………………………………… I am a Research Assistant with the Institute of Human Virology, Nigeria working as part of a project called EQUAL which seeks to improve maternal and newborn health outcomes. We are currently conducting a research study on Midwifery Education in Conflict-Affected Areas of Nigeria. The study seeks to provide deeper understanding and actionable insights into issues influencing midwives' ability to deliver quality MNH services findings. Findings will help inform programmatic recommendations that will potentially benefit midwifery students and educators and result in a stronger local health workforce in the future. This part of the study will assess the extent to which midwifery pre-service education programs in EQUAL study locations meet national and global (International Confederation of Midwives) standards. It will also explore how conflict affects pre-service midwifery education in EQUAL study locations.

This study will involve asking you some questions about your impressions and experiences with this midwifery program. Participation is completely voluntary – you can choose not to participate, and even if you agree to participate, you may stop at any time. Any information we collect is confidential. We will not record your names and no personal individual information will be shared with anyone.
There will be no direct benefit to you from participating in this study. We are asking for your help to collect information to improve midwifery education in Nigeria.

If you wish to participate in the study, I will now proceed to ask you a series of questions.

May I continue?

If "no" → Thank you for your time and have a nice day.

In case you need more information about the survey, you may contact the person listed on this card [PROVIDE CARD OR PAPER WITH STUDY CONTACT].

INTRODUCTION (Somalia)

Good Morning/Afternoon/Evening: My name is Mr. /Mrs. /Ms. /Dr……………………………………… I work with the Somali Research and Development Institute (SORDI) working as part of a project called EQUAL which seeks to improve maternal and newborn health outcomes. We are currently conducting a research study on Midwifery Education in Conflict-Affected Areas of Somalia. The study seeks to provide deeper understanding and actionable insights into issues influencing midwives' ability to deliver quality MNH services findings. Findings will help inform programmatic recommendations that will potentially benefit midwifery students and educators and result in a stronger local health workforce in the future. This part of the study will assess the extent to which midwifery pre-service education programs in EQUAL study locations meet national and global (International Confederation of Midwives) standards. It will also explore how conflict affects pre-service midwifery education in EQUAL study locations.
This study will involve asking you some questions about your impressions and experiences with this midwifery program. Participation is completely voluntary – you can choose not to participate, and even if you agree to participate, you may stop at any time. Any information we collect is confidential. We will not record your names and no personal individual information will be shared with anyone.

There will be no direct benefit to you from participating in this study. We are asking for your help to collect information to improve midwifery education in Somalia.

If you wish to participate in the study, I will now proceed to ask you a series of questions.
May I continue?

If "no" → Thank you for your time and have a nice day.

In case you need more information about the survey, you may contact the person listed on this card [PROVIDE CARD OR PAPER WITH STUDY CONTACT].

For MOH officials, provost, health professional associations and UN agencies

Midwifery education standards		
**1.1**	**Is there an organization that has been designated the responsibility for the regulation of midwifery practice?**	`□ Yes<br>□ No<br><br>If yes, please specify:
**1.2**	**Is the same regulatory agency responsible for setting quality standards for midwifery education?**	`□ Yes<br>□ No<br><br>Please explain:
**1.3**	**Is there a process for obtaining inputs from midwives on these standards?**	`□ Yes<br>□ No<br><br>Please explain:
Accreditation		
**1.4**	**Is there an organization that has been designated the responsibility for the accreditation of midwifery education programs?**	`□ Yes<br>□ No<br><br>Please explain:
**1.5**	**Is there a mechanism or program established for conducting reviews of midwifery education program quality or performance?**	`□ Yes<br>□ No<br><br>If yes, how often is each program reviewed?
Licensing		
**1.6**	**Is there a system of licensing for midwives before they start practicing?**	`□ Yes<br>□ No<br><br>Please explain:
**1.7**	**Does the system include some measure of assessment of theoretical knowledge and/or clinical competence as a condition of licensure?**	`□ Yes<br>□ No<br><br>If yes, who is responsible for developing teaching methods?<br><br>What are minimum passing criteria?
**1.8**	**Is the license to practice given for life, or is there criterion for re-assessment at a later time, including evidence of clinical practice**	□ Given for life<br>□ Reassessment (retesting) required for re-licensure<br>□ Continuing education units required for re-licensure

	**and/or for accumulation of continuing education credits prior to re-licensure**	
Financing		
**1.9**	**Does the government have a committed budget for sustaining midwifery education needs?**	`□ Yes<br>□ No<br><br>Please explain:
**1.10**	**Is the budget sufficient for year-to-year operation of midwifery education programs?**	□ Yes<br>□ No<br><br>Please explain:
**1.11**	**Are there external donors supporting midwifery program operation expenses** (including teacher salaries, supplies, program operating costs)**?**	□ Yes<br>□ No<br><br>Please explain:
Gender and social inclusion		
**1.12**	**Do midwifery education programs prepare students for gender inequities they may face in the workplace?**	□ Yes<br>□ No<br><br>Please explain:
**1.13**	**Do midwifery education programs include training on how to care for women affected by gender based violence?**	□ Yes<br>□ No<br><br>Please explain:
**1.14**	**Do midwifery educations include training on how to care for women with disabilities?**	□ Yes<br>□ No<br><br>Please explain:
Insecurity and emergency preparedness		
**1.15**	**Do midwifery education programs include training on how to work in settings affected by conflict and security?**	□ Yes<br>□ No<br><br>Please explain:
**1.16**	**Do midwifery education programs include training on midwifery services during disease outbreaks?**	□ Yes<br>□ No<br><br>Please explain:
Program quality		
**1.17**	**How has insecurity affected midwifery education**	

	**programs in this area?**	
	**Do you think students currently enrolled in midwifery education programs will be well prepared to work as midwives after graduation?**	□ Yes<br>□ No<br><br>Please explain:

For local government officials and community leaders

**1.1**	**After their graduation, what type of support, if any, do community midwives get from community leaders?**	Please describe:
**1.2**	**Do community midwives run into any problems with families in the community?**	□ Yes<br>□ No<br><br>Please explain:

Thank the key informant for their time.

**Interview end time**	
